# Rapamycin attenuates pathological hypertrophy caused by an absence of trabecular formation

**DOI:** 10.1038/s41598-018-26843-1

**Published:** 2018-06-05

**Authors:** Nicole D. Fleming, Leigh A. Samsa, David Hassel, Li Qian, Jiandong Liu

**Affiliations:** 1 Department of Pathology and Laboratory Medicine, University of North Carolina at Chapel Hill, Chapel Hill, NC 27519 USA; 2 Department of Cell Biology and Physiology, University of North Carolina at Chapel Hill, Chapel Hill, NC 27519 USA; 3 McAllister Heart Institute, University of North Carolina at Chapel Hill, Chapel Hill, NC 27519 USA; 4 Department of Internal Medicine III-Cardiology, Im Neuenheimer Feld 350, University of Heidelberg, Heidelberg, 69120 Germany

## Abstract

Cardiac trabeculae are mesh-like muscular structures within ventricular walls. Subtle perturbations in trabeculation are associated with many congenital heart diseases (CHDs), and complete failure to form trabeculae leads to embryonic lethality. Despite the severe consequence of an absence of trabecular formation, the exact function of trabeculae remains unclear. Since ErbB2 signaling plays a direct and essential role in trabecular initiation, in this study, we utilized the *erbb2* zebrafish mutant as a model to address the function of trabeculae in the heart. Intriguingly, we found that the trabeculae-deficient *erbb2* mutant develops a hypertrophic-like (HL) phenotype that can be suppressed by inhibition of Target of Rapamycin (TOR) signaling in a similar fashion to adult mammalian hearts subjected to mechanical overload. Further, cell transplantation experiments demonstrated that *erbb2* mutant cells in an otherwise wildtype heart did not undergo hypertrophy, indicating that *erbb2* mutant HL phenotypes are due to a loss of trabeculae. Together, we propose that trabeculae serve to enhance contractility and that defects in this process lead to wall-stress induced hypertrophic remodeling.

## Introduction

The function of the heart is intimately linked to its structure. In order to meet an increasing physiological demand of the growing embryo, the developing vertebrate heart undergoes extensive chamber topological remodeling to increase total cardiac output. Most notably is the formation of cardiac trabeculae, the mesh-like luminal projections within ventricular myocardium^1–6^. Cardiac trabeculation is a complex and tightly regulated morphogenetic process that involves cardiomyocyte (CM) apical constriction followed by CM depolarization and remodeling of myocardial cell-cell adhesion^7,8^. These highly coordinated cellular events ultimately lead to CM delamination and emergence of trabeculae^2^. Despite substantial progress in our understanding of the cellular and molecular basis of cardiac trabeculation, the exact function of trabeculae in the heart remains unclear. Cardiac trabeculation is essential for life, as subtle perturbations in trabeculation are associated with many congenital heart diseases (CHDs), and complete failure to form trabeculae leads to embryonic lethality across different species^2,9–11^. Yet, how loss of trabeculae leads to a lethal phenotype remains an open question.

Although the fundamental cellular and morphological changes associated with cardiac trabeculation occur mostly in the myocardium, this process requires crosstalk at the molecular level between endocardial and myocardial cells^2,3,5,12–14^. Nrg/ErbB signaling constitutes one of the most important signaling pathways required for cardiac trabeculation, and is a key node for this crosstalk. Neuregulins, expressed on endocardial cells, are part of the epidermal growth factor receptor ligand family and signal to the myocardial cells through its ErbB4-ErbB2 receptor complex and are essential for trabeculation in multiple model systems^5,13,15,16^. Mouse embryos deficient of *Nrg1*, *ErbB2* or *ErbB4* all fail to form trabeculae^11^. Likewise, loss of Nrg/ErbB2 signaling in zebrafish embryos results in a complete absence of trabecular formation^2^. By taking advantage of the unique attributes of zebrafish embryos, Liu *et al*., demonstrated that pharmacological inhibition of ErbB2 signaling right before cardiac trabeculation completely blocked this process from occurring, suggesting an immediate and direct effect of Nrg/ErbB2 signaling on trabecular formation. As such, the zebrafish *erbb2* mutant is advantageous to investigate the cellular and functional consequences when the heart loses its normal internal cardiac trabecular structure.

In this study, we found that *erbb2* mutant ventricles exhibit an increase in ventricular cardiomyocyte cross-sectional area and myofibril size. This cardiac phenotype is reminiscent of hypertrophic growth of an adult mammalian heart subjected to mechanical overload. Consistently, we found that the expression of hypertrophic marker gene, *nppa*, was elevated in *erbb2* mutants compared to controls. Intriguingly, inhibition of Target of Rapamycin (TOR) signaling by rapamycin suppressed *erbb2* mutant hypertrophic-like (HL) growth phenotypes and rescued cardiac function. Additionally, cell transplantation experiments indicate the *erbb2* mutant HL phenotypes are due to a loss of cardiac trabeculae. Together, our findings suggest that trabeculae serve to enhance contractility for efficient cardiac function and that defects in this process lead to wall-stress induced pathological hypertrophic remodeling.

## Results

### *erbb2* mutant hearts exhibit hypertrophic-like phenotypes

**Figure 1:**
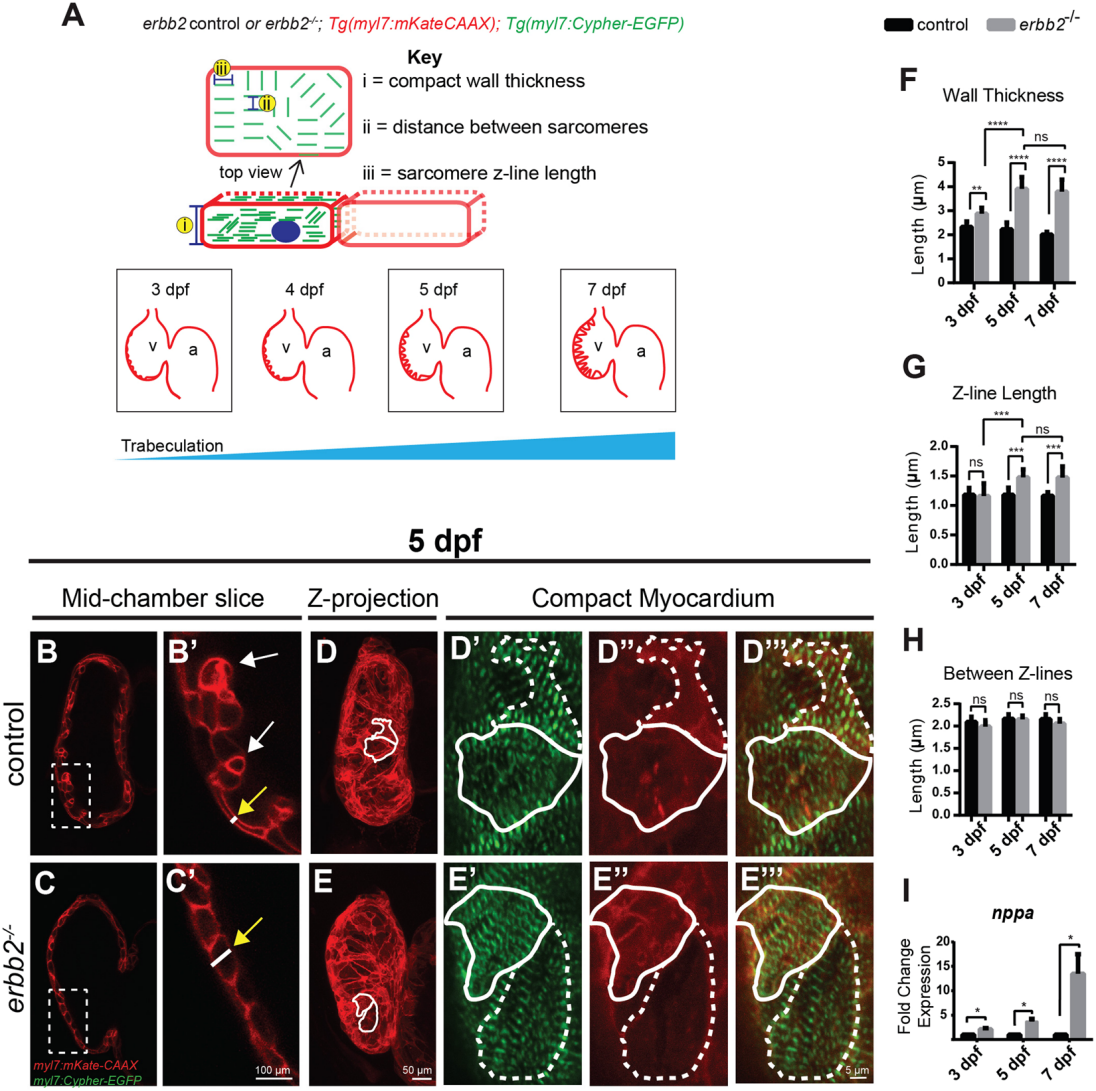
*erbb2* mutant develops HL phenotypes (**A**) Schematic of method utilized for obtaining compact myocardial wall and myofibril measurements. (**B**,**C**) Mid-chamber confocal sections of control and *erbb2*^*−/−*^ hearts, respectively. (**B’**,**C’**) Magnified high-resolution images of compact myocardial wall and trabecular regions marked by dotted box in B, C. Yellow arrows point to length of CM along compact myocardial wall. White arrows point to trabeculae. (**D**,**E**) Maximal projection of confocal Z-stacks of ventricle. (**D’**-**D”’**, **E’**-**E”’**) Magnified high-resolution images of compact myocardium outlined in white in D and E revealing sarcomere structures of two CMs in control and *erbb2*^*−/−*^ ventricles, respectively. (**F**–**H**) Quantification of compact myocardial wall thickness (n = 6–10), Z-line length (n = 9–10), and distance between Z-lines (n = 8–10), at 3, 5, and 7 dpf from control and *erbb2*^*−/−*^ hearts. (**I**) Expression of *nppa* in whole hearts isolated from *erbb2* control or *erbb2*^*−/−*^ larvae (n = 10–15). Data are represented as mean ± SEM. *p ≤ 0.05, **p = 0.0025, ***p = 0.0004 and ****p < 0.0001 by Student’s t test. v, ventricle; a, atrium.

While the embryonic heart requires cardiac contraction to initiate trabecular formation^17,18^, failure of cardiac trabeculation could cause the developing heart to suffer not only structural defects, but also mechanical disturbance that might lead to further myocardial damages. We have previously reported that larvae homozygous for the *erbb2*^*st61*^ allele encoding a premature stop codon, from here on referred to as *erbb2*^*−/−*^ or *erbb2* mutants, develop progressive systolic and diastolic dysfunction^2^. The reduction in fractional shortening in *erbb2* mutant hearts observed in Liu *et al*.^2^ is a probable consequence of the loss of trabeculation, suggesting that cardiac trabeculae are important for proper cardiac function and overall workload efficacy of the ventricle. To evaluate the underlying cardiomyocyte defects associated with the cardiac dysfunction observed in *erbb2* mutant hearts, we bred the *erbb2*^*st61*^ allele onto *Tg(myl7:mKateCAAX)* and *Tg(myl7:Cypher-EGFP)* transgenic backgrounds to label the CM plasma membrane and Z-band of cardiac sarcomere, respectively (Fig. 1A).

Using these genetically encoded fluorescent protein labels, we assessed cardiomyocyte structure in *erbb2*^*−/−*^ larvae by confocal microscopy compared to control larvae carrying at least one copy of the wildtype *erbb2* allele. This technique allowed us to measure the outer compact myocardial wall thickness, distance between sarcomeres, and Z-line length at the trabeculation stages (Fig. 1A). When comparing outer compact myocardial wall, we observed a significant, albeit rather mild, increase in the myocardial wall thickness in *erbb2* mutant ventricles compared to control ventricles at 3 days post fertilization (dpf), when trabeculae are small in the ventricle (Supplementary Fig. S1A-1B’). At 5 and 7 dpf, when the control heart becomes extensively trabeculated, the difference in the outer compact myocardial wall thickness between the two genotypes was even greater. For instance, at 5 and 7 dpf, the outer compact myocardial wall thickness increased by 75% and 88% in *erbb2* mutant ventricles relative to control ventricles, respectively (Fig. 1B-C’,F; Supplementary Fig. S1E-F’). In contrast, it only increased by 23% at 3 dpf (Fig. 1F). Since the zebrafish ventricular compact myocardial wall is one-cell thick^19^, the compact myocardial wall thickness is equivalent to compact CM cross-sectional area. We also measured Z-band size as an indicator of ventricular sarcomere thickness. Cardiac remodeling in response to mechanical overload also includes the addition of sarcomeres in parallel to existing sarcomeres^20^. Consistent with the compact wall thickness phenotype, Z-band length increased in the *erbb2* mutant hearts by 5 dpf. Though comparable at 3 dpf, with control hearts, ventricular sarcomere thickness of *erbb2* mutant hearts became significantly increased on 5 dpf and 7 dpf by 26.2% and 27.6%, respectively (Fig. 1D-E”’,G; Supplementary Fig. S1C-D” and 1G-H”). Suggesting that sarcomeric function is normal, and that the sarcomeres are structurally intact, the distance between Z-bands was unchanged in *erbb2* mutants (Fig. 1H). Taken together, these data suggest that *erbb2* mutant ventricles that lack trabeculae exhibited an increase in ventricular CM cross-sectional area and myofibril size, a phenotype indicative of the cellular changes that underlie biomechanical overload-induced cardiac hypertrophy^21^.

Next, we addressed whether the HL phenotypes exhibited by *erbb2* mutant were pathological. At the molecular level, pathological cardiac hypertrophy is typically associated with upregulation of marker genes, such as *natriuretic peptide precursor A* (*nppa*)^22–24^. *nppa* is conserved in zebrafish and its embryonic expression has been well characterized^25^. During cardiac development, as cardiac trabeculae start to form, *nppa* expression is down-regulated in the ventricular compact myocardium, and becomes restricted in cardiac trabeculae^26^. To evaluate whether *nppa* was upregulated in *erbb2* mutants, we first selected putative *erbb2* mutants based on a supernumerary neuromast phenotype detectable by 3 dpf with vital dye MitoTracker. *nppa* expression was observed in *erbb2* control hearts (Supplementary Fig. S1I-I’). Interestingly, though *erbb2* mutant ventricles are completely devoid of cardiac trabeculae, whole mount *in situ* hybridization revealed robust *nppa* expression in the ventricular compact myocardium (Supplementary Fig. S1J-J’). More importantly, RT-PCR analysis of isolated control and mutant hearts demonstrated that the expression of *nppa* was dramatically upregulated in *erbb2* mutants compared to controls (Fig. 1I), suggesting a pathological nature of *erbb2* mutant hypertrophic phenotypes.

### Inhibition of TOR signaling attenuates *erbb2* mutant hypertrophic-like phenotype

**Figure 2:**
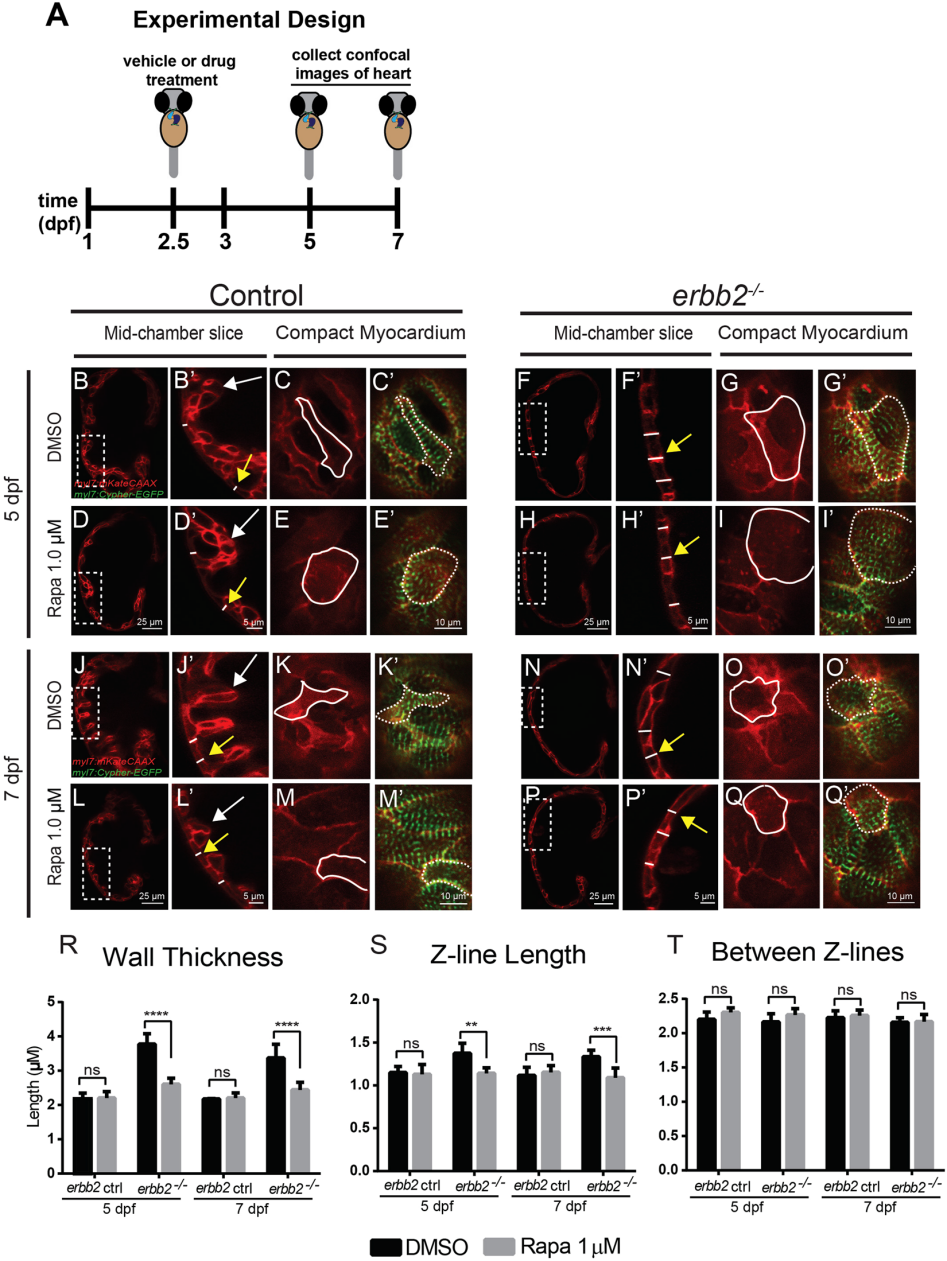
Inhibition of TOR signaling attenuates *erbb2* mutant HL phenotypes (**A**) Schematic of experimental design. (**B**,**D**,**F**,**H**,**J**,**L**,**N**,**P**) Mid-chamber confocal sections of control and *erbb2*^*−/−*^ hearts treated with DMSO or rapamycin at 5 and 7 dpf. (**B’**,**D’**,**F’**,**H’**,**J’**,**L’**,**N’**,**P’**) Magnified high-resolution images of compact myocardial wall and trabecular regions marked by dotted box in (B,D,F,H,J,L,N,P). Yellow arrows point to length of CM along compact myocardial wall. White arrows point to trabeculae. (**C-C’**,**E-E’**,**G-G’**,**I-I’**) Magnified high-resolution images of compact myocardium revealing sarcomere structures of CMs at 5 dpf. (**K-K’**,**M-M’**,**O-O’**,**Q-Q’**) Magnified high-resolution images of compact myocardium revealing sarcomere structures of CMs at 7 dpf. (**R–T**) Quantification of compact myocardial wall thickness (n = 7–15), Z-line length (n = 5–10), and distance between Z-lines (n = 5–12) at 5 and 7 dpf from DMSO or rapamycin-treated control and *erbb2*^*−/−*^ hearts. Data are represented as mean ± SEM. **p = 0.0035, ***p = 0.0003, and ****p < 0.0001 by Student’s t test.

To identify signaling pathways essential for *erbb2* mutants to develop HL phenotypes, we performed a targeted small molecule screen to inhibit signaling pathways known to be involved in regulating cardiac hypertrophy^20,27–30^. We treated control and *erbb2* mutant larvae carrying plasma membrane and sarcomere reporters with 1% DMSO or small molecules at 2.5 dpf, just prior to emergence of the first trabeculae, then assessed ventricular compact wall thickness and cardiac sarcomere structure at 5 and 7 dpf (Fig. 2A). Inhibition of TOR signaling with rapamycin at a concentration of 1 µM suppressed the increased compact myocardial wall thickness and Z-band length phenotypes in *erbb2* mutant at both 5 dpf (Fig. 2B-I’ and R-S) and 7 dpf (Fig. 2J-Q’ and R-S). Narrowing in on the TOR pathway, we observed similar changes to compact myocardial wall thickness and myofibril length when TOR was inhibited via 0.5 µM Rapamycin (Supplementary Fig. S2A–C) or 0.5 µM Torin 1 (Supplementary Fig. S2D–F), another potent inhibitor of the TOR pathway. Notably, rapamycin treatment did not cause any compact myocardium and sarcomere defects in control hearts, nor did it have any effect on distance between Z-lines in the mutant hearts (Fig. 2T). Together, our data strongly indicates inhibition of TOR signaling by rapamycin treatment suppressed *erbb2* mutant HL phenotypes. Thus, *erbb2* mutant hearts develop hypertrophy in a TOR-dependent manner.

### Rapamycin treatment improves impaired heart function in *erbb2* mutant

**Figure 3:**
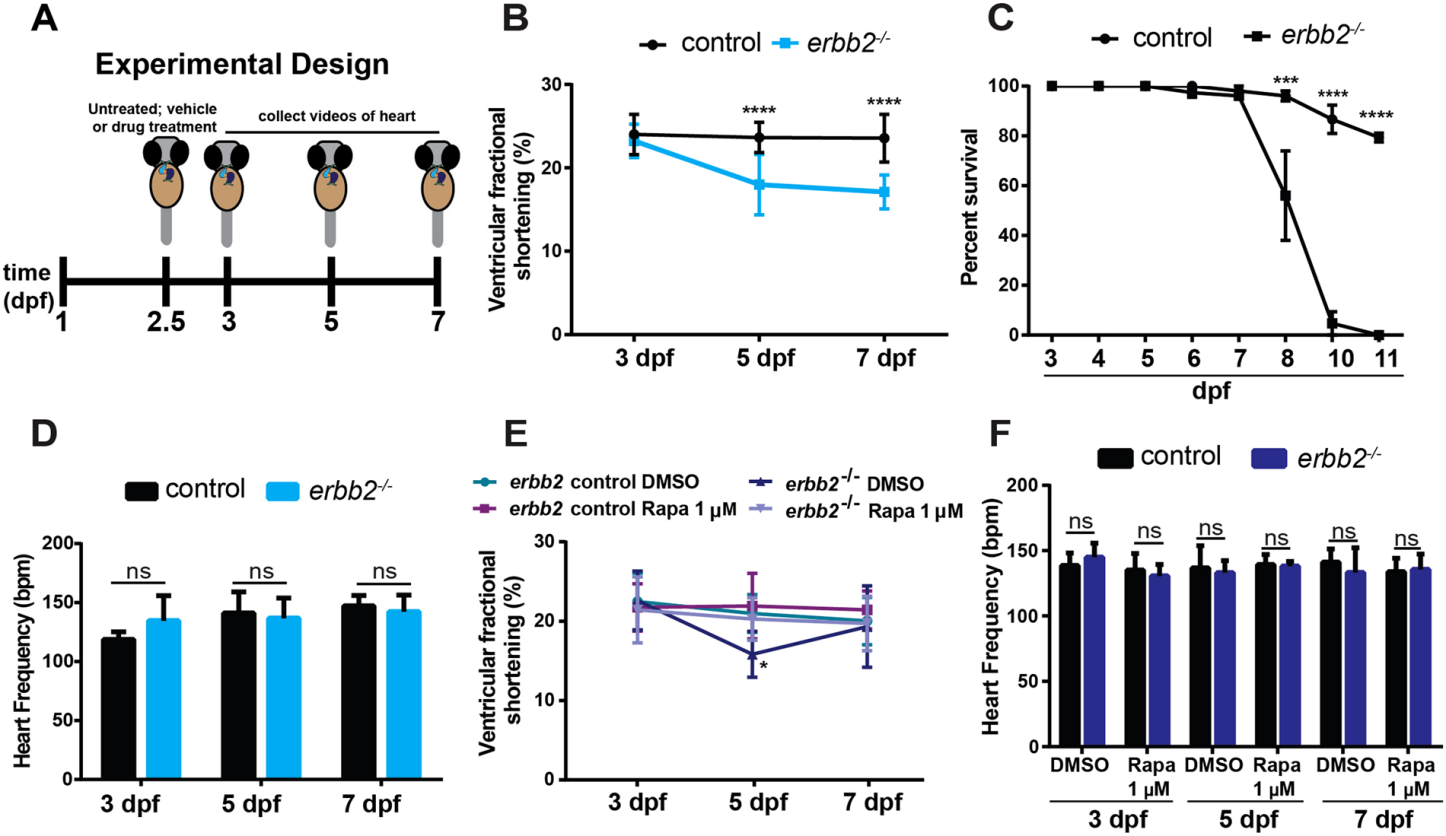
Rapamycin treatment improves impaired heart function in *erbb2* mutant (**A**) Schematic of experimental design. (**B**) Quantification of ventricular fractional shortening of control and *erbb2*^*−/−*^ larvae at 3, 5, and 7 dpf (n = 10). (**C**) Quantification of percent survival over ~2 weeks of control and *erbb2*^*−/−*^ clutchmates from N = 3 tanks of 50 embryos each. (**D**) Quantification of heart rate in beats per minute of control and *erbb2*^*−/−*^ larvae at 3, 5, and 7 dpf (n = 9–10). (**E**) Quantification of ventricular fractional shortening of DMSO or rapamycin-treated control and *erbb2*^*−/−*^ larvae at 3, 5, and 7 dpf (n = 9–16). (**F**) Quantification of heart rate in beats per minute of DMSO or rapamycin-treated control and *erbb2*^*−/−*^ larvae at 3, 5, and 7 dpf (n = 4–11). Data are represented as mean ± SEM. *p = *0*.*0499*, ***p ≤ *0*.*001*, and ****p < 0.0001 by Student’s t test. Note: Asterisk in E indicates a significant difference from 5 dpf rapamycin–treated *erbb2*^*−/−*^. No significant differences were observed between groups on 3 or 7 dpf.

The above experiments indicate that inhibition of TOR signaling by rapamycin suppressed HL phenotypes in *erbb2*^*−/−*^ larvae hearts. We next wanted to test if this attenuation of HL phenotypes translated into an enhancement or even further deterioration of cardiac function (Fig. 3A). Consistent with previous studies^2^, we found that *erbb2* mutants exhibited progressive cardiac dysfunction. At 3 dpf, the ventricular fractional shortening (FS) of *erbb2* mutants is comparable to that of control animals, but the mutant displayed significantly reduced FS at 5 dpf, which declined even further at 7 dpf (Fig. 3B). Consequently, *erbb2* mutant animals started to die at 7 dpf (Fig. 3C). However, no significant difference on heart rate was observed between control and *erbb2* mutants (Fig. 3D). Interestingly, upon treatment with rapamycin from 2.5 dpf onwards, the *erbb2* mutant hearts displayed significantly improved ventricular FS at 5 dpf compared to DMSO-treated *erbb2* mutant hearts (*p* = *0*.*0499)*. Though, this improvement in heart function is not sustainable over time (Fig. 3E). Furthermore, we did not notice any significant difference in heart rate between DMSO and rapamycin-treated groups (Fig. 3F). Together, these findings suggest that the TOR pathway is an important regulator of the HL phenotype and inhibition of TOR signaling delayed disease progression of the *erbb2* mutant hearts.

### *erbb2* mutant HL phenotype results from the absence of trabecular formation

**Figure 4:**
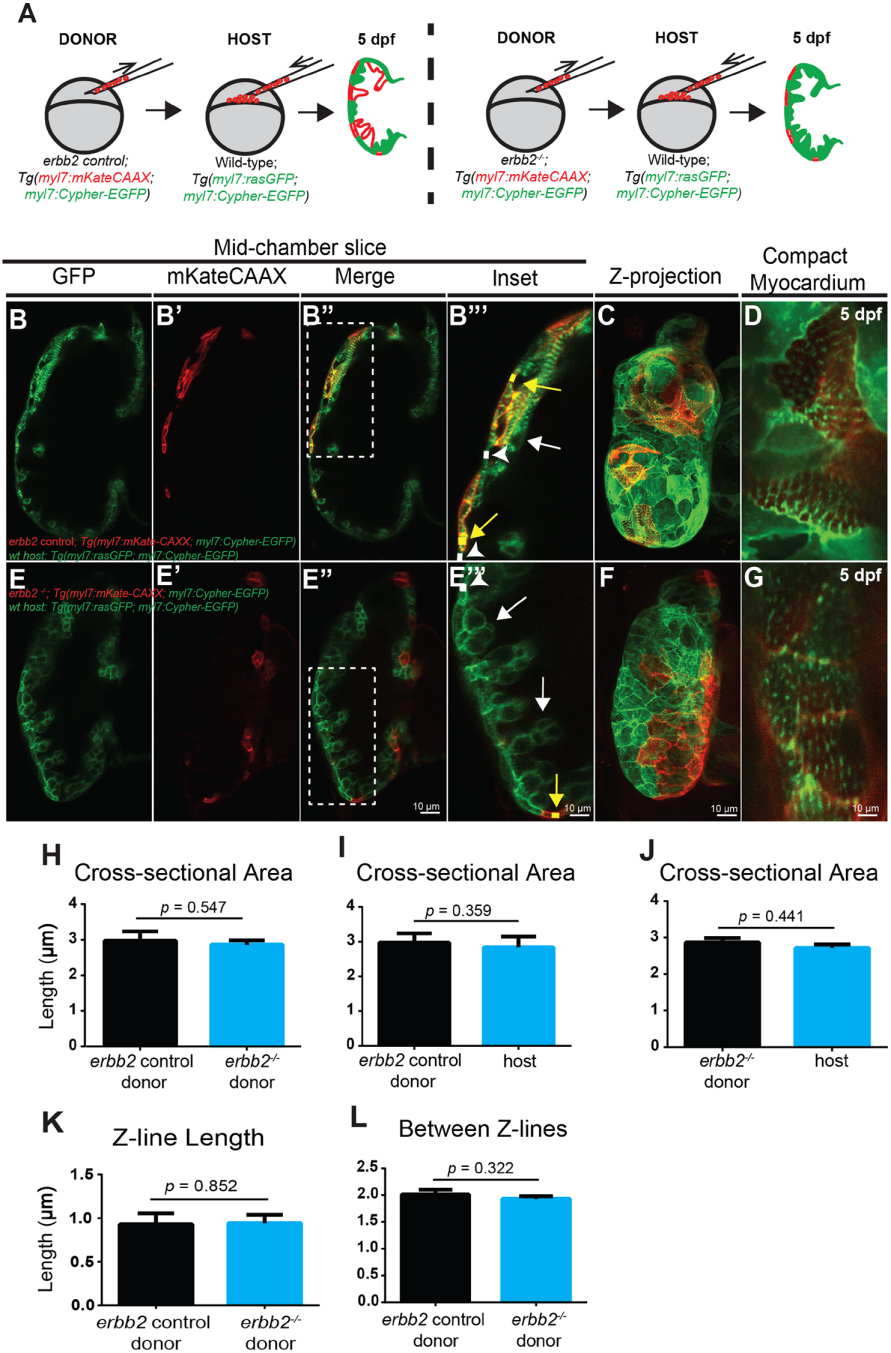
*erbb2* mutant HL phenotypes result from the absence of trabecular formation (**A**) Schematic of transplantation experiment resulting in transplanted blastomeres from *Tg(myl7:mKateCAAX);Tg(myl7:Cypher-EGFP) erbb2* control or *erbb2*^*−/−*^ donors into *Tg(myl7:rasGFP); Tg(myl7:Cypher-EGFP)* wild-type hosts. (**B**-**B”**,**E**-**E”**) Mid-chamber confocal sections of control and *erbb2*^*−/−*^ donor CMs, respectively, in wild-type host ventricle. (**B”’**,**E”’**) Magnified high-resolution images of compact myocardial wall and trabecular regions marked by dotted box in B”, E”. Yellow arrows point to length of donor CM (yellow line) along compact myocardial wall. White arrow heads point to length of host CM (white line) along compact myocardial wall. White arrows point to trabeculae. (**C**,**F**) Maximal projection of confocal Z-stacks of ventricle. (**D**,**G**) Magnified high-resolution images of compact myocardium, revealing sarcomere structures of control and *erbb2*^*−/−*^ donor CMs, respectively, in wild-type host ventricle at 5 dpf. (**H**–**J**) Quantification of cross-sectional area of *erbb2* control donor and *erbb2*^*−/−*^ donor CMs (**H**), *erbb2* control donor and host CMs (**I**), and *erbb2*^*−/−*^ donor and host CMs at 5 dpf (**J**). (**K**) Quantification of Z-line length of *erbb2* control donor and *erbb2*^*−/−*^ donor CMs at 5 dpf. (**L**) Quantification of distance between Z-lines of *erbb2* control donor and *erbb2*^−/−^ donor CMs at 5 dpf. Data are represented as mean ± SEM.

As the *erbb2* mutant HL phenotypes are reminiscent of hypertrophic growth of an adult heart subjected to mechanical overload^21^, we hypothesize that these HL phenotypes could be due to mechanical disturbance as a result of the absence of trabecular formation. Yet, it is also possible that the HL phenotypes could result from a loss of *erbb2* function cell-autonomously in the cardiomyocytes. To distinguish these possibilities, we generated genetic mosaic embryos whereby *erbb2* mutant or control cells were transplanted into wildtype embryos. The host embryos were allowed to develop until 5 dpf before monitoring CM cross-sectional area and myofibril size of the transplanted cells. In these experiments, we transplanted blastomeres from *erbb2*^*−/−*^ or control donors that carried *Tg(myl7:mKateCAAX)*, *Tg(myl7:Cypher-EGFP)* transgenes into *Tg(myl7:rasGFP*)*; Tg(myl7:Cypher-EGFP)* hosts. Transplanted donor cardiomyocytes were labeled in red by *Tg(myl7:mKateCAAX)*, and thus, were readily distinguishable from the host derived cells (Fig. 4A). Noteworthy, the transplanted mKateCAAX-positive control cells populated both the compact and trabecular layers (Fig. 4B-B”’). The mKateCAAX-positive *erbb2* mutant cells failed to contribute to the trabeculae and were found exclusively in the compact layer (Fig. 4E-E”’). Most importantly, we found that the cross-sectional area of the transplanted *erbb2* mutant cardiomyocytes was not significantly different from that of the transplanted control cardiomyocytes (Fig. 4B-B”’,E-E”’ and H) and their neighboring compact cardiomyocytes (Fig. 4I-J). Similarly, there was no significant difference in Z-line length (Fig. 4C-D,F-G and K) or the distance between Z-lines (Fig. 4L) between transplanted control and *erbb2* mutant cardiomyocytes. Thus, these data suggest that ErbB2 does not directly regulate compact ventricular myocardial wall thickness, and that *erbb2* mutant HL phenotype is caused by the absence of trabecular formation.

## Discussion

**Figure 5:**
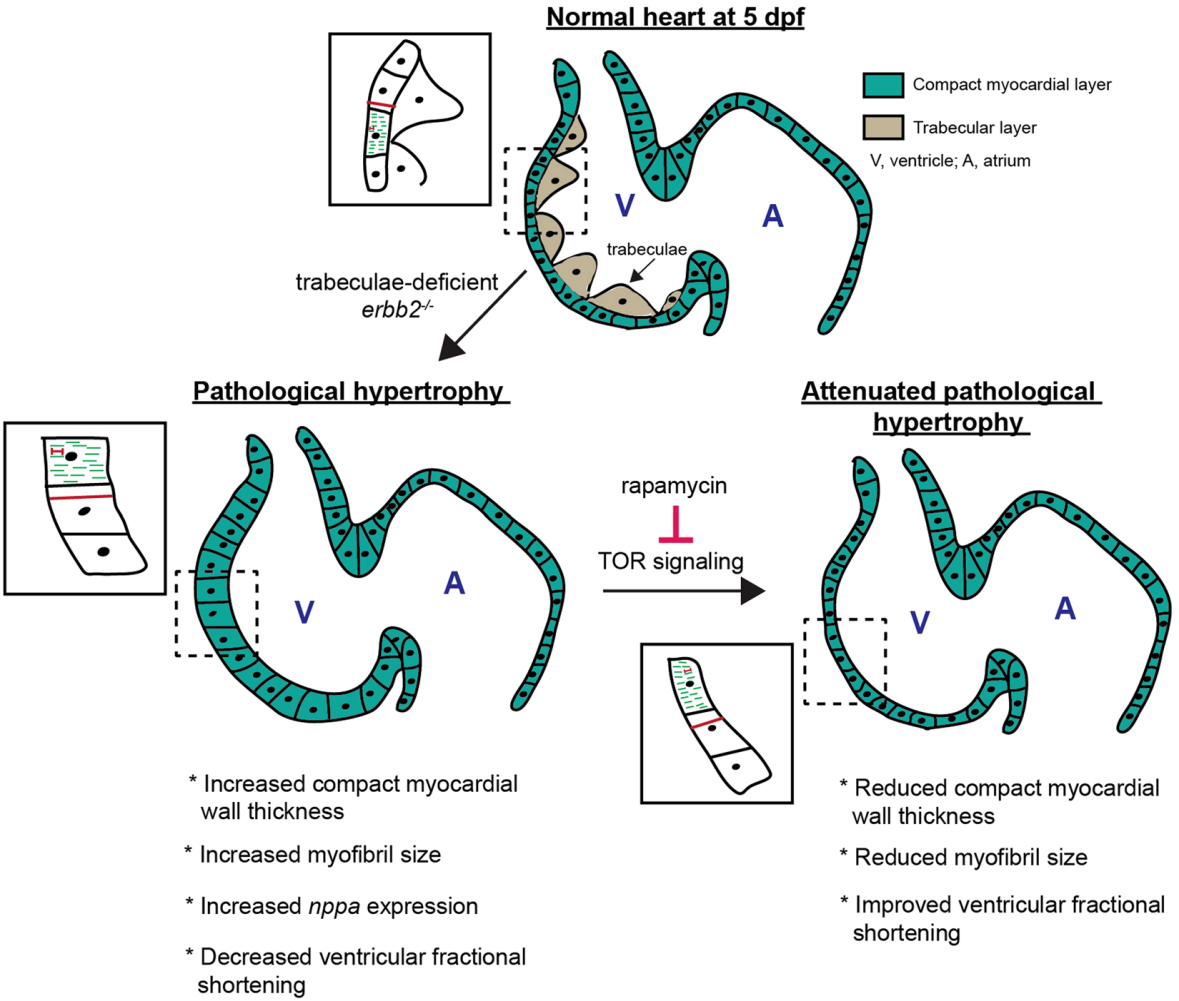
Schematic diagram illustrating that trabeculae-deficient *erbb2* mutants undergo TOR-dependent pathological hypertrophy.

In this study, we sought to investigate the molecular mechanisms that impact the remodeling process when the heart loses its normal internal cardiac trabecular structure. We present *in vivo* evidence that cardiac trabeculae have an important physiological function during embryonic heart development. Interestingly, we found *erbb2* mutant ventricles exhibit an increase in ventricular CM cross-sectional area and myofibril size, likely due to an absence of trabecular formation (Fig. 5), suggesting that trabeculae-deficient ventricles undergo hypertrophy to stabilize the workload/cardiac output imbalance in the heart.

It has been speculated that cardiac trabeculae serve to increase cardiac output and facilitate the exchange of oxygen and nutrients prior to formation of the coronary vasculature^1,2,5,6^. Nevertheless, there have been very few studies that examine exactly how trabeculae help the heart function in an efficient manner. Mice lacking *Nrg1*, *ErbB4*, or *ErbB2* fail to form trabeculae and thus die at approximately E10.5^11^. The early embryonic lethality of these systems disallows for detailed characterization of the trabeculae-deficient hearts at a cellular level. Zebrafish embryos do not require a functional circulatory system during the first week of life and thus can survive very severe cardiac defects^31,32^. Thus, we took advantage of the zebrafish *erbb2* mutant that lacks trabeculae to decipher the consequence of an absence of trabecular formation and found that trabeculae-deficient hearts exhibited HL phenotypes reminiscent of those of an adult mammalian heart subjected to mechanical overload. As an organism grows, the heart undergoes morphological changes to meet the demand of increase workload. Our findings thus suggest that trabeculae serve to enhance contractility and that defects in this process lead to wall-stress induced pathological hypertrophic remodeling.

Pathological cardiac hypertrophy is induced by mechanical pressure-overload^21^. The intracellular signaling activated by the mechanical stress imposed on the myocardium induces extensive structural and physiological changes to offset the mechanical stress. Currently, there are no viable methods to directly measure the intraventricular radial wall stress “felt” by cardiomyocytes. To circumvent this gap of knowledge, future studies are needed to focus on using 3D computational models of the function in the zebrafish heart to extract quantitative assessments of radial wall stress. Specifically, numerical simulations can be used to compare radial wall stresses generated during contraction of the control and *erbb2* mutant hearts to readily obtain spatial and temporal distributions of radial wall stresses.

Recent studies have identified multiple cell signaling pathways to be involved in cardiac hypertrophy, including TOR^20,27–30^. Signaling through the TOR pathway has been shown to be an important regulator of normal cardiac growth and pathological hypertrophy^21,27^. We examined signaling pathways that could attenuate the hypertrophic response in *erbb2* mutant hearts. Our small-molecule pharmacological studies indicated that inhibition of TOR signaling suppressed *erbb2* mutant HL growth phenotype (Fig. 5). The TOR pathway has been implicated as a potential therapeutic target for cardiomyopathies because inhibition by rapamycin, a specific inhibitor of TORC1, has been shown to be cardio-protective^33,34^. In human patients, rapamycin treatment following heart transplantation significantly reduced left ventricular hypertrophy^35^. Intriguingly, in mice, pharmacological administration of rapamycin prevents cardiac hypertrophy induced by transverse aortic constriction and reverses hypertrophic cardiomyopathy in a model of CHD LEOPARD syndrome^27^. These findings suggest a common pathological signaling pathway of CHD-associated hypertrophic cardiomyopathy across zebrafish, mouse and humans.

In this present study, we also provide evidence suggesting that *erbb2* mutant hearts undergo pathological hypertrophy probably due to mechanical overload because they lack trabeculae rather than to deficiency in ErbB2 signaling. Our study cannot completely rule out the possibility that HL phenotypes are manifested as a consequence of ErbB2 deficiency and are independent of the absent of trabecular formation. In fact, previous studies have shown that various trabeculation-deficient zebrafish embryos that have impaired flow and cardiac contractions display distinct differences in CM behavior and formation of protrusions^3,7,17,36^. Because of the transparency of the zebrafish embryos and the ease of genetic manipulation, the unique HL phenotype caused by the absence of trabeculae in ErbB2-deficient larvae will provide us a great opportunity to use zebrafish as a model to assess molecular events and mechano-transduction signaling pathways involved in CHD-associated hypertrophic cardiomyopathy. In addition, the small size and permeability of zebrafish embryos also enable high-throughput small molecule screens to identify novel suppressors of disease phenotypes. The zebrafish *erbb2* mutant thus could serve as an *in vivo* platform for phenotype-based drug screen to identify novel small molecules capable of suppressing CHD-associated hypertrophic cardiomyopathy.

## Materials and Methods

### Zebrafish husbandry and strains

All animals were maintained at the aquaculture facility of the University of North Carolina at Chapel Hill in accordance with Institutional Animal Care and Use Committee approved protocols^37^. We used the following transgenic and mutant lines: *erbb2*^*st61*^ ^38^ mutants, *Tg(myl7:mKateCAAX)*^*sd11*^ ^39^, *Tg(myl7:Cypher-EGFP)*^*xu061*^ ^40^, *Tg(myl7:ras-GFP)*^*s883*^ ^19^.

### Confocal imaging and analysis

Zebrafish embryos were anesthetized using 1X Tricaine (Sigma) and mounted with 1% low-melting agarose (Sigma) in embryo medium or system water for optimal visualization of the heart. Heartbeat was ceased before imaging using 5–10X Tricaine (Sigma). Images were obtained with an Olympus Fluoview 1000MPE, multiphoton, upright confocal microscope equipped with a 20X XLPlan (NA 1.0) water immersion objective with 3.0X, 2.5X, or 10X digital zoom and 1024 × 1024 pixels resolution. Confocal z-stacks were collected using the Fluoview software with a 2.50 µm or 1.05 µm step size acquisition through the heart tissue. 488 nm and 599 nm lasers were used to excite GFP and RFP. Each channel was acquired sequentially to avoid bleed through and signal attenuation due to increasing depth was taken into account by using the software’s brightness correction algorithm. Confocal images were collected for a minimum of 5 embryos for each condition. Data was analyzed using ImageJ^41^ software.

### Compact myocardial wall and sarcomere measurements

ImageJ^41^ software was used to analyze confocal z-stacks of heart tissue. For ventricular compact myocardial length, a line (total of 12 lines) was drawn from one end of the plasma membrane to the next of individual cardiomyocytes through different mid-chamber z-stacks to obtain an average measurement from a minimum of 5 embryos. An average measurement of distance between sarcomeres was obtained by drawing a line (N = 20) from the middle of one Z-line to the middle of the neighboring Z-line for a minimum of 5 embryos. To obtain sarcomere Z-line length, Z-lines of individual cardiomyocytes were outlined and average length of Z-lines and area of cardiomyocytes were recorded for a minimum of 5 cardiomyocytes.

### Cardiac function measurements

Embryos were genotyped using MitoTracker Red (Life Technologies) and immobilized with 0.5X  Tricaine (Sigma). Embryos were mounted using 1% low-melting agarose (Sigma) and oriented for optimal observation of hearts at 3, 5, and 7 dpf. After agarose solidified, embryos were incubated with fresh egg water for 20 minutes to remove any residual Tricaine and renew normal cardiac contraction. A Leica M205C fluorescence stereomicroscope and software were used to visual and record live imaging of *Tg(myl7:mKateCAAX)*; *Tg(myl7:Cypher-EGFP)* positive hearts at 120X or 200X magnification. For each condition, heartbeats per minute were obtained from 15-second movies. Additional details are as previously described^39^. Assessment of fractional shortening was performed as previously described^2^. Briefly, for each embryo, the average diameter at end systole (fully contracted ventricle) and end diastole (fully dilated ventricle), during three cardiac cycles, was obtained using VirtualDub software (available at http://www.virtualdub.org) and ImageJ^41^.

### Survival curve

Zebrafish embryos from interbreeding *erbb2*^+/−^ *Tg(myl7:mKateCAAX); Tg(myl7:Cypher-EGFP)* fish were screened at 3 dpf using MitoTracker Red to identify *erbb2* control and mutant embryos. Survival was recorded daily from N = 3 tanks of 50 embryos each per genotype.

### Chemical inhibition of hypertrophic pathways

Pharmacological inhibitors were stored in DMSO (Fisher Scientific) stocks. Embryos were treated with drugs, 0.5 µM or 1 µM rapamycin (Santa Cruz Biotech) or 0.5 µM Torin 1 (Cayman Chemical), diluted in egg water (1.5 mL stock salts added to 1 L distilled water) and supplemented with 0.003% 1-phenyl 2-thiourea (PTU) (Sigma) and DMSO to 1% final. Control embryos were incubated with 1% DMSO vehicle. Embryos were dechorionated and treated with DMSO, rapamycin or Torin 1 at 2.5 dpf until 5 dpf and 7dpf.

### Whole heart isolations and quantitative RT-PCR

At 3, 5, and 7 dpf, zebrafish embryos were euthanized and hearts were isolated with forceps using a Leica M205C fluorescence stereomicroscope and placed in lysis buffer prior to RNA isolation. At least 10 hearts were pooled per sample and RNA was extracted using a RNeasy Plus Mini kit (Qiagen). cDNA synthesis was performed immediately following RNA isolation protocol using iScript cDNA synthesis kit (Invitrogen). Quantitative RT-PCR was performed using cDNA, Sybr Green reagent (Roche) and gene-specific primers in triplicates using an Applied Biosystem Viia7 RT-PCR machine (Life Technologies). The primer sequences used in this study are provided in Supplementary Table S1. The delta-delta CT method was used to normalize desired genes to the endogenous housekeeping gene *vmhc* and determine the relative fold change gene expression.

### Whole-mount *in situ* hybridization

*In situ* hybridization was performed as previously described^42,43^. Briefly, genotyped zebrafish embryos were fixed with 4% paraformaldehyde in 1X PBS at 5 dpf and placed in 4 °C cold room overnight. Embryos were then washed in 1X PBS (3 × 10 minutes), incubated for 30 minutes in 100% methanol then stored in 100% methanol at −20 °C. Zebrafish embryos were rehydrated in PBST and incubated in proteinase K (20 mg/ml diluted in PBT 2:2000) for 45 minutes then post-fixed in 4% PFA. The tail of *erbb2* mutant embryos were removed using fine forceps, and both control and mutant embryos were placed in the same tube to ensure consistent handling throughout the staining process. Hybridization with probe of interest was done overnight in a 65–70 °C water bath. Following washes, embryos were incubated with anti-DIF-AP antibody (Roche) overnight at 4 °C. Subsequent color development was achieved following addition of NBT/BCIP. Embryos were then stored in stop solution at 4 °C overnight. Next, embryos were transferred to 100% glycerol and placed at 4 °C overnight. Embryos were mounted in 100% glycerol for imaging. Whole-mount embryos were visualized using a Leica MZ16F fluorescence stereomicroscope with Retiga 2000R camera and images were collected using the QCapture software.

### Cell Transplantation

Mosaic embryos were obtained using *erbb2* control or mutant *Tg(myl7:mKateCAAX)*; *Tg(myl7:Cypher-EGFP)* and *Tg(myl7:ras-GFP);Tg(myl7:Cypher-EGFP*) double transgenic lines. Cells were removed from donor embryos and transplanted into the marginal zone of stage-matched host embryos. Embryos were visualized using a Leica S6D microscope. Cells were transplanted using borosilicate thin wall capillary glass tubing needles (Warner Instruments) and a CellTram vario (Eppendorf) apparatus that allowed for precise control of pressure transmission with oil. Host embryos with mKateCAAX-expressing cells were allowed to develop until 5 dpf. Donor embryos from in-crossing *erbb2*^*st61*^ heterozygotes were kept to determine genotype of donor cells as previously described^38^. Embryos were mounted for imaging, as previously described^17^, for observing compact myocardial wall and sarcomere measurements.

### Statistical Analysis

The data shown in the graphs are represented as mean ± SEM. Statistical significance was determined between groups with a two-tailed Student’s t-test.

## Electronic supplementary material

Supplementary Information
