# Supplementary material for: Rapamycin attenuates pathological hypertrophy caused by an absence of trabecular formation
Source: Sci Rep. 2018 Jun 5;8:8584. doi: 10.1038/s41598-018-26843-1 (PMC5988815; doi:10.1038/s41598-018-26843-1)
Supplement: Supplementary file 1 — Supplementary Information [file 41598_2018_26843_MOESM1_ESM.docx]

**Rapamycin attenuates pathological hypertrophy caused by an absence of trabecular formation**

Nicole D. Fleming,^1,3^ Leigh A. Samsa,^2,3^ David Hassel,^4^ Li Qian,^1,3^ Jiandong Liu^1,3*^

^1^Departments of Pathology and Laboratory Medicine, ^2^Cell and Molecular Physiology, ^3^McAllister Heart Institute, University of North Carolina at Chapel Hill, Chapel Hill, NC, 27519, USA

^4^Department of Internal Medicine III-Cardiology, Im Neuenheimer Feld 350, University of Heidelberg, Heidelberg 69120, Germany

*Corresponding author. Tel: +1 919 962 0326; E-mail: [Jiandong_liu@med.unc.edu](mailto:Jiandong_liu@med.unc.edu)

**
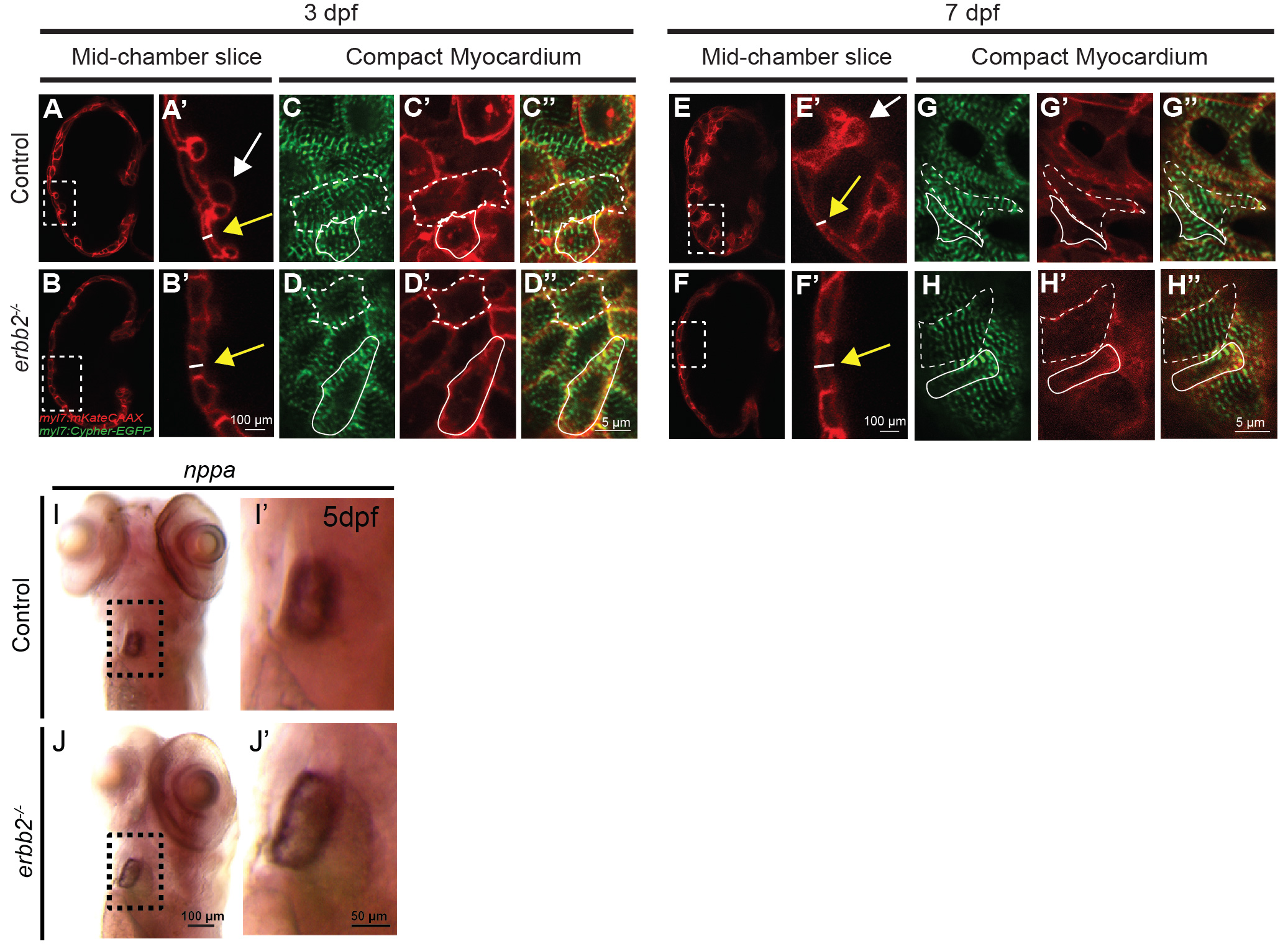
Figure S1. *erbb2* mutant exhibits more pronounced HL phenotype overtime.**

(A,B,E,F) Mid-chamber confocal sections of *erbb2* control and *erbb2^-/-^* hearts at 3 and 7 dpf. (A',B',E',F') Magnified high-resolution images of compact myocardial wall and trabecular regions marked by dotted box in A,B,E,F. Yellow arrows point to length of CM (white line) along compact myocardial wall. White arrows point to trabeculae. (C-C'',D-D'',G-G",H-H") Magnified high-resolution images of compact myocardium, revealing sarcomere structures of two CMs. (I, J) Representative images of whole-mount *in situ* hybridization of *nppa* in *erbb2* control and *erbb2^-/-^* hearts, respectively. (I’, J’) Magnified images of heart region marked by dotted box in I and J. (n = 12).

**
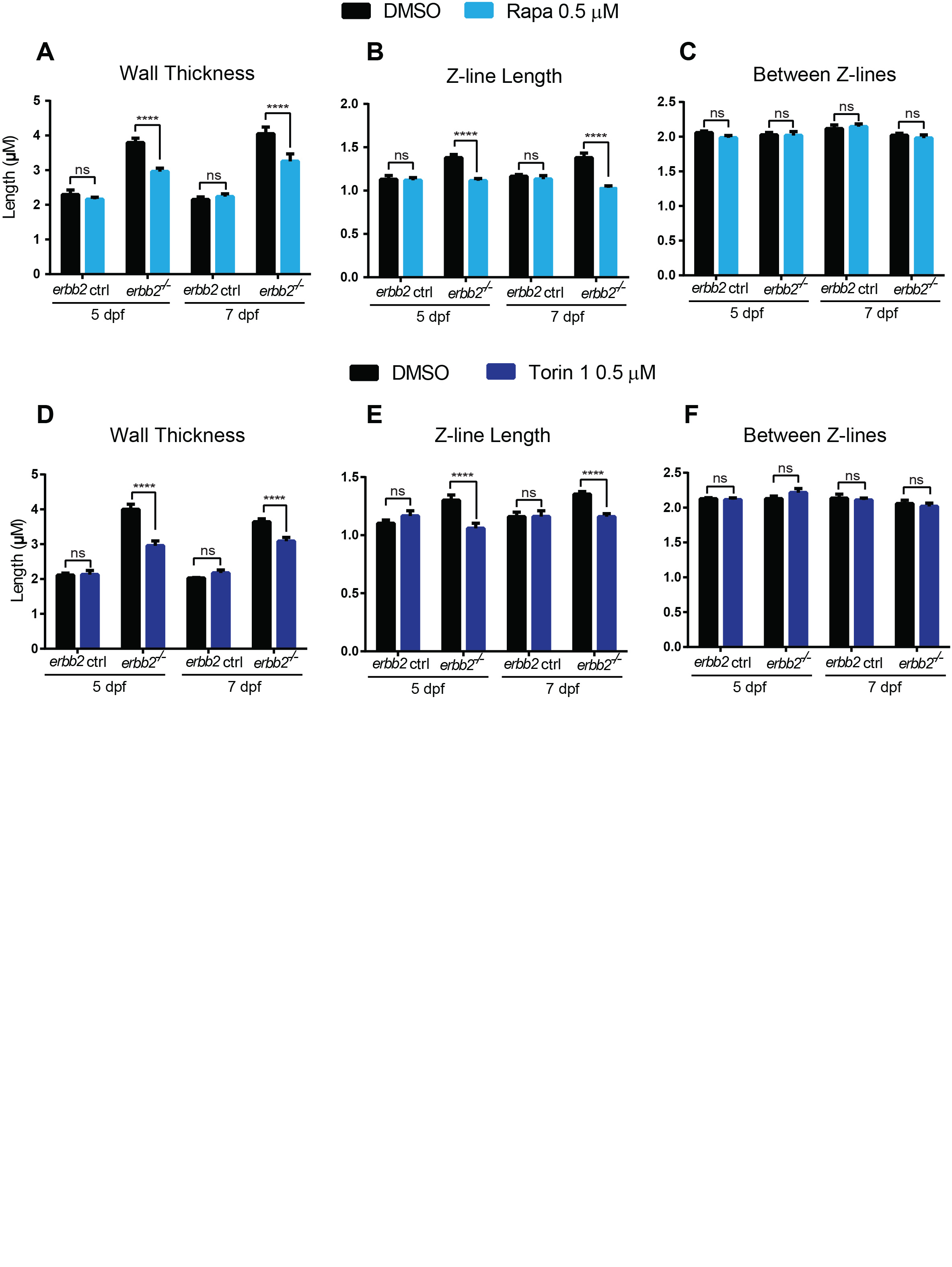
Figure S2. Inhibition of TOR signaling with lower concentration of Rapamycin and Torin1 attenuates *erbb2* mutant HL phenotypes.**

(A-C) Quantification of compact myocardial wall thickness (n = 3-8), Z-line length (n = 4-9), and distance between Z-lines (n = 4-8) at 5 and 7 dpf from DMSO or (0.5 µM) rapamycin-treated *erbb2* control or *erbb2*^-/-^ hearts. (D-F) Quantification of compact myocardial wall thickness (n = 3-8), Z-line length (n = 5-8), and distance between Z-lines (n = 3-8) at 5 and 7 dpf from DMSO or (0.5 µM) Torin 1-treated *erbb2* control or *erbb2^-/-^* hearts. Data are represented as mean ± SEM. *****p* < 0.0001 by Student's t test.

**Table S1. qRT-PCR primer sequences**

| Gene | Forward (5'-3') | Reverse (3'-5') |
| --- | --- | --- |
| *vmhc* | GAGACGAACAACCTTCTGC | AGCAAGCTTACGGCCTCTTT |
| *nppa* | CTGGTTTGGCAGCAGACGGATG | GGCCTCCTCAAACTGCTGCA |
